# Supplementary material for: Repeatability of Inertial Measurements of Spinal Posture in Daily Life
Source: Sensors (Basel). 2025 Aug 13;25(16):5011. doi: 10.3390/s25165011 (PMC12390158; doi:10.3390/s25165011)
Supplement: Supplementary file 1 [file sensors-25-05011-s001.zip › sensors-3746963-supplementary.pdf]

Supplementary 1: Sensor-Specific Errors

The repeatability of the standardized tasks is affected by the natural variation in the performance of the tasks by the participants, and it is impossible to separate variation caused by sensor related factors from the person related factors in this experiment. To investigate issues that are specifically related to the sensors and processing techniques, we carried out an additional data collection in which 3 IMUs were strapped to a box for 2 days and carried around through daily life. The sensor settings and data processing of the IMU data were the same as in the main experiment.

Because all IMUs were attached to the same rigid object, each IMU is expected to output the same estimates for angles (assuming no error). To account for any differences of how the sensors were attached to the box, the Kabsch algorithm was used to impose alignment between all sensors prior to further processing. The relative difference in angle between each pair of sensors was used as the metric for error. The absolute value of the error between sensors was averaged across time and across each plane of motion.

We assessed the extent to which clock resampling (Brønd et al., 2021)), gyroscope bias correction, and magnetic interference affected error. Across the whole experiment, we found that gyroscope bias correction and the exclusion of magnetic interference had the largest effects on error, whereas the clock resampling correction had a smaller effect (Fig. S2_1). Error reduction for gyroscope bias correction, clock resampling, and exclusion of magnetic interference decreased error by 1.9°, 0.4°, and 1.0°, respectively. Relative to the baseline error rate of 5.8°, these changes represented reductions in error of 33%, 7%, and 17%, respectively. Although the clock resampling had a smaller relative effect in this additional data collection, we generally observed greater desynchronization between sensors in the main experiment. This discrepancy could in part be explained by the sensors being attached directly to humans and generally going through a greater

variety of environments which, for example, could have different temperatures, a factor which affects clock timing.

Although the results for this data collection used averaged data from across the entire experiment, only a small portion of the data contained magnetic interference. When looking at specifically the periods of disturbance, it can be observed that magnetic interference severely influences error, increasing error on average from 2.4° (with no disturbance) to 14.2° (with both sensors disturbed), an increase of 478% (Fig. S2_2).

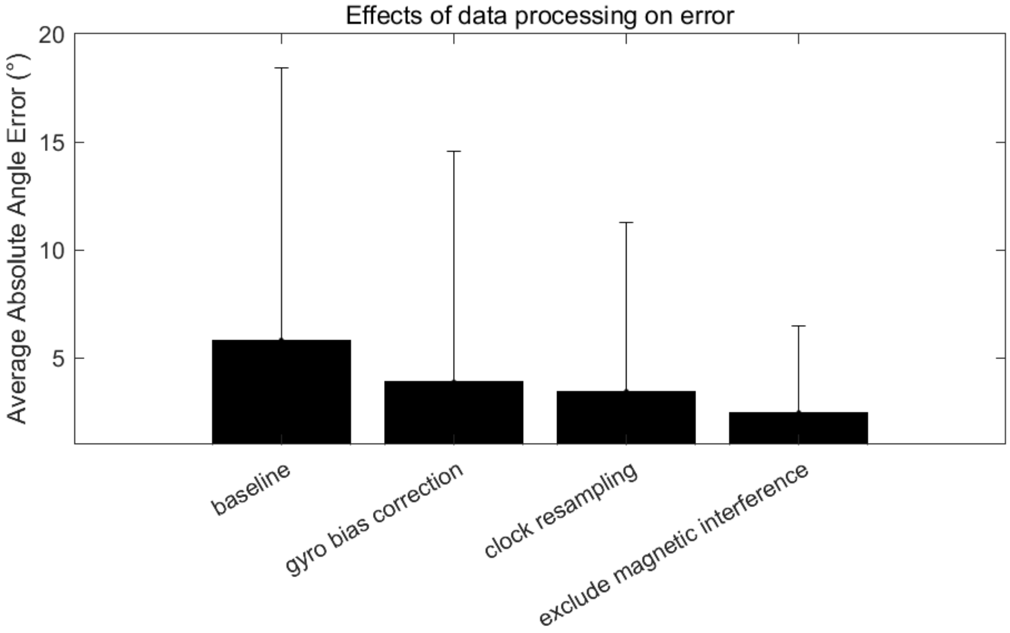

Figure S1_1. Effects of different data processing techniques on the error in measurement of IMU orientation estimation across the experiment. From left to right, each subsequent processing improvement were included such that each estimate includes the cumulative effects of each prior modification. Error bars show standard deviation.

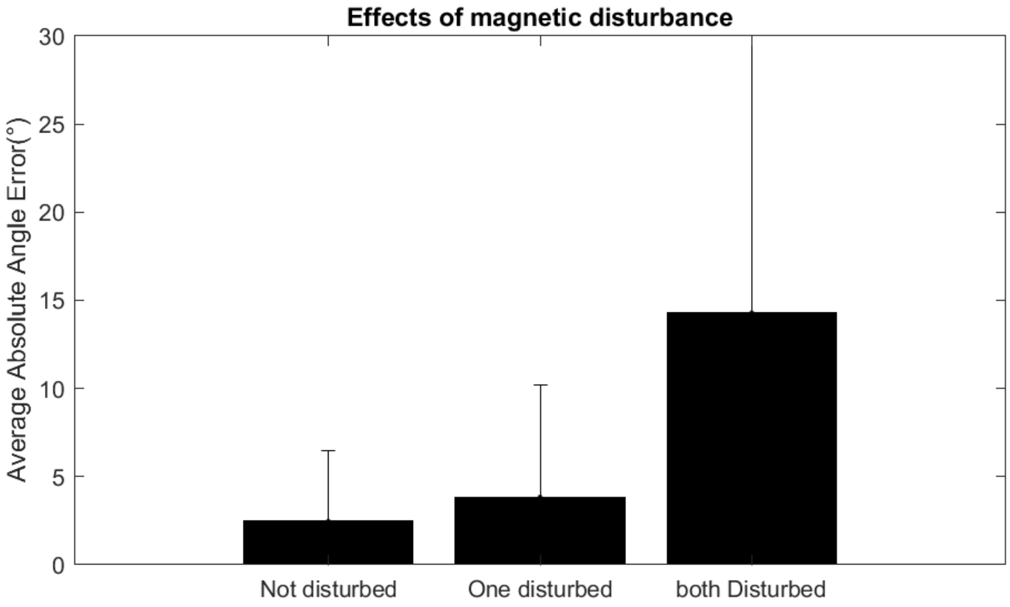

Figure S1_2. Effect of magnetic disturbance on error when comparing the angle estimates of two sensors. The bars (from left to right) show the average error across all angles when neither sensor was disturbed, one sensor was disturbed, or both were disturbed. Error bars show standard deviation.

Although only a case study, these data reinforce the need for careful identification of magnetic interference. Note that the average error with all such corrections applied was 2.4°, which is comparable to a previous estimate of IMU error of 2.2° found by the same IMUs in a laboratory context measuring human motions at 20 Hz (Riddick et al., 2023). Although this experiment did not directly validate the accuracy of human measurements during daily living, it highlights the importance of identification and use of precise techniques for data processing specific to the sensors to identify and/or manage any factors that may cause data artifacts in any recordings with IMUs.

References

Brønd, J.C., Pedersen, N.H., Larsen, K.T., Grøntved, A., 2021. Temporal Alignment of Dual Monitor Accelerometry Recordings. Sensors 21, 4777. https://doi.org/10.3390/s21144777

Riddick, R., Smits, E., Faber, G., Shearwin, C., Hodges, P., van den Hoorn, W., 2023. Estimation of human spine orientation with inertial measurement units (IMU) at low sampling rate: How low can we go? J. Biomech. 157, 111726. https://doi.org/10.1016/j.jbiomech.2023.111726
